# Automated object recognition in high-resolution optical remote sensing imagery

**DOI:** 10.1093/nsr/nwad122

**Published:** 2023-05-04

**Authors:** Yazhou Yao, Tao Chen, Hanbo Bi, Xinhao Cai, Gensheng Pei, Guoye Yang, Zhiyuan Yan, Xian Sun, Xing Xu, Hai Zhang

**Affiliations:** School of Computer Science and Engineering, Nanjing University of Science and Technology, China; School of Computer Science and Engineering, Nanjing University of Science and Technology, China; Aerospace Information Research Institute, Chinese Academy of Sciences, China; School of Electronic, Electrical and Communication Engineering, University of Chinese Academy of Sciences, China; Key Laboratory of Network Information System Technology (NIST), Aerospace Information Research Institute, Chinese Academy of Sciences, China; School of Computer Science and Engineering, Nanjing University of Science and Technology, China; School of Computer Science and Engineering, Nanjing University of Science and Technology, China; Department of Computer Science and Technology, Tsinghua University, China; Aerospace Information Research Institute, Chinese Academy of Sciences, China; School of Electronic, Electrical and Communication Engineering, University of Chinese Academy of Sciences, China; Key Laboratory of Network Information System Technology (NIST), Aerospace Information Research Institute, Chinese Academy of Sciences, China; Aerospace Information Research Institute, Chinese Academy of Sciences, China; School of Electronic, Electrical and Communication Engineering, University of Chinese Academy of Sciences, China; Key Laboratory of Network Information System Technology (NIST), Aerospace Information Research Institute, Chinese Academy of Sciences, China; School of Computer Science and Engineering, University of Electronic Science and Technology of China, China; Pazhou Laboratory (Huangpu), China; School of Mathematics, Northwest University, China; Pazhou Laboratory (Huangpu), China

## Abstract

This paper reports the background and results of the Automated Object Recognition in Optical Remote Sensing Imagery, which is one of the tracks in 2022 International Algorithm Case Competition, as well as summarize the challenges, champion solutions, and future directions.

## INTRODUCTION

With the rapid development of remote sensing technology, high-quality remote sensing images have become widely accessible. The automated object detection and recognition of these images, which aims to automatically locate objects of interest in remote sensing images and distinguish their specific categories, is an important fundamental task in the field. It provides an effective means for geo-spatial object monitoring in many social applications, such as intelligent transportation, urban planning, environmental monitoring and homeland security.

Actually, automated interpretation of remote sensing data is a challenging task due to the wide space coverage and complicated image background. Traditional methods mainly focus on manually designed features to represent objects of interest, which may limit the model performance. Recently, with the growing wave of deep learning, methods based on the convolutional neural network (CNN) have made great progress in this field. However, it still has difficulty in some specific tasks such as densely distributed small object detection and requires highly advanced techniques.

### Challenge

Specifically, as shown in Fig. S1 within the online supplementary material, automated object detection and recognition in remote sensing images has the following challenges.

1. **Large variation in object sizes**. The wide coverage leads to multi-class objects in remote sensing scenes. While different classes of objects may vary greatly in size. For example, the area of a standard football field is 7140 square meters, while a car is only about 10 square meters. Thus, multi-scale features are important in this case.
2. **Dense distribution of small objects**. There are a large number of vehicles, ships and other small objects arranged in a dense distribution in the remote sensing images, resulting in a high missed alarm rate of the model.
3. **Low signal-to-noise ratio**. The imaging quality of some remote sensing images may be poor due to the influence of clouds, fog, etc. Some interesting objects may be blurred or submerged in noise, which is difficult to locate.

In order to promote academic research in this field, this competition focuses on the task of automated object detection and recognition in high-resolution remote sensing imagery. The competitors are required to accurately locate the position of multi-class objects and identify their specific categories with oriented bounding boxes.

### Competition details

#### Dataset

The FAIR1M-1.5 dataset provided by the Aerospace Information Research Institute, Chinese Academy of Sciences is used in this competition. The images are collected from the Gaofen-2 satellite and Google Earth with resolutions ranging from 0.3–0.8 m and sizes ranging from 600–2000 pixels. The scenes cover more than 50 airports, ports and urban areas around the world. There are 10 categories in the dataset: airplane, ship, vehicle, basketball court, tennis court, football field, baseball field, intersection, roundabout, bridge. In the competition, the dataset is divided into three parts: 5000 samples for training, 576 samples for testing in the preliminary stage and 577 samples for testing in the final stage.

#### Evaluation metric

We adopt the mean average precision (mAP) as the evaluation metric in this competition. The calculation of mAP follows the Pascal VOC 2012 Challenge [1].

## THE SOLUTION OF THE CHAMPION TEAM

**Figure 1.**
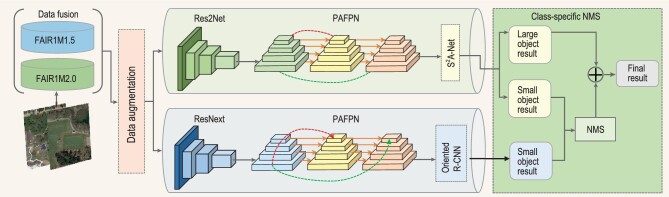
The overall architecture of the proposed approach by the champion team.

This section introduces the winner’s solution. The overall architecture of the proposed approach is shown in Fig. 1. We first fuse the training dataset provided by the competition (i.e. FAIR1M-1.5) with another public dataset FAIR1M-2.0 [2]. Then, data augmentation is applied to the mixed dataset. We adopt the single-stage detector $\rm S^2A$-Net[3] and the two-stage detector Oriented R-CNN [4] to detect the objects in remote sensing images with Res2Net [5] and ResNext [6] as the backbones, respectively. Features from these backbones are further enhanced by path aggregation feature pyramid network (PAFPN) [7]. After obtaining the detection results, we propose the class-specific non-maximum suppression (NMS) to fuse the results from two detectors according to the object size of categories.

### Mix-grained data fusion

This competition provides 5000 remote sensing images with 10 coarse-grained categories as the training dataset. Meanwhile, FAIR1M-2.0 [2] is a much larger benchmark dataset with 16 488 training images for fine-grained object recognition in high-resolution remote sensing imagery. Whereas images in FAIR1M-2.0 [2] and the training dataset provided by this competition are captured with the same device under similar scenarios, the two datasets have similar data distributions. Therefore, making good use of FAIR1M-2.0 [2] can benefit our object detection task in remote sensing images.

Though the transfer learning strategy has been widely adopted to learn from additional data and then transfer knowledge to the target dataset, it will involve parameter fine-tuning and classifier adjustment due to the different number of dataset categories. Considering the category relevance and the similarity of data distribution between FAIR1M-2.0 [2] and the competition training dataset, we propose to group all 37 subcategories in FAIR1M-2.0 [2] into 10 defined categories in the competition. Specifically, we transform all the instance annotations in the training and validation sets of FAIR1M-2.0 [2] into coarse-grained categories and merge them with the competition dataset for joint training.

### Data augmentation

To alleviate the difficulties in the remote sensing detection task, we employ the following data augmentation strategies.

Since objects are presented at arbitrary angles in remote sensing images, we adopt random rotation augmentation (i.e. 90°, 180°, 270°) and random flip (i.e. horizontal and vertical flips) to provide the same instance with different rotation angles, which aims to improve the angle robustness of the learned model.

We note that the competition training dataset and FAIR1M-2.0 [2] have long-tail distribution problems. Specifically, the head class of Vehicle accounts for 74% of all instance annotations, while seven tail categories only take up 4%. Therefore, we resample instances of each tail category to a certain number to mitigate the long-tail effect.

Besides, color space distribution in remote sensing images is distinctively different from that in ImageNet. Therefore, we calculate the mean and variance of the mixed training dataset to replace the original settings in ImageNet.

We also apply Mixup for data augmentation to improve the generalization ability of the model.

### Model ensemble with class-specific NMS

#### Feature extraction

As a powerful technique to increase accuracy, the model ensemble has been widely adopted in various competitions. In this approach, a single-stage detector $\rm S^2A$-Net[3] and a two-stage detector Oriented R-CNN [4] are trained for the model ensemble. For feature extraction, Res2Net [5] and ResNext [6] are adopted as the backbones of the one-stage $\rm S^2A$-Net [3] detector and the two-stage Oriented R-CNN [4] detector, respectively. Then we further exploit the multi-scale features to get enhanced feature representations. Though the traditional FPN excels at fusing higher-level semantic information with its multi-layer feature extraction network, it tends to lose the low-level location information. Therefore, we resort to PAFPN [7] that combines FPN with a bottom-up path augmentation. It shortens the information path between the lower layers and the top feature and preserves precise location information in the underlying features.

#### Class-specific NMS

While $\rm S^2A$-Net [3] proposes aligning axis-aligned convolutional features and arbitrary-oriented objects with alignment convolution, Oriented R-CNN [4] predicts the minimum bounding rectangle and the offsets instead of directly predicting the rotated proposal box to avoid the angle boundary problem. Though both detectors show superior detection performance on oriented object detection, their accuracy on different scale categories shows significant differences due to their varying model structures.

Therefore, we propose a class-specific NMS module to exploit the merits of the two models for improving the detection capability of the ensemble. On the one hand, $\rm S^2A$-Net [3] with aligned convolutional features has excellent detection capability for arbitrary scale objects. On the other hand, Oriented R-CNN [4] has more advantages in detecting small object categories like Vehicle and Ship due to its capability of producing high-quality oriented proposals in the first stage. Hence, for these classes with mainly small-scale objects, we fuse the detection results from two detectors through NMS. For the medium-scale and large-scale objects, the detection results of $\rm S^2A$-Net [3] are used directly since $\rm S^2A$-Net performs better than Oriented R-CNN [4].

### Implementation details

The development of the solution and the training of models in this competition are based on the Jittor [8] deep learning framework.

During training, the original remote sensing images are first resized at three scales (0.5, 1.0 and 1.5), and then cropped to a series of 1024 × 1024 pixel patches with a stride of 200 pixels. The model used in this competition is trained with eight NVIDIA RTX3090 GPUs (two images per GPU) for 30 epochs with an initial learning rate of 0.01, decreasing it by 0.1 after 20 and 25 epochs.

Test time augmentations like flipping (horizontal and vertical) and multi-scale testing are exploited during the testing phase. Furthermore, the NMS is applied with a 0.1 IoU threshold.

### Results

In this section, the results of the proposed solution to this competition are reported. As shown in Table S1 within the online supplementary material, the solution achieves the top-1 mAP in both the preliminary and final rounds of the competition. The visualization of the detection results from the champion’s solution is shown in Fig. S2 within the online supplementary material. The model has achieved good performance in several challenging remote sensing scenes, which all demonstrate the effectiveness and robustness of the method.

The element-wise component analysis is demonstrated in Table S2 within the online supplementary material. As can be seen, with our adopted data augmentation, we can improve the baseline result from 75.63 to 77.52 mAP. In our experiments, we note that transfer learning with fine-grained data only brings about a 0.4 mAP performance gain. In contrast, with our proposed mix-grained data fusion strategy, we can further enhance the accuracy by 2.6 mAP, which is the most important component for performance improvement. Moreover, our proposed model ensemble with class-specific NMS can yield another 1 mAP performance gain, and the mAP finally reaches 81.16.

Besides, the efficiency of the proposed solution is also quantitatively compared in terms of model size and inference speed. As shown in Table S3 within the online supplementary material, due to the model ensemble, the efficiency of the solution is slightly lower than that of a single model. But the average inference time of each image with 1024 × 1024 pixels is about 0.5 s, which can meet the requirements of some practical applications.

### COMMENTS ON THE CHAMPION’S SOLUTION

The champion’s solution proposes a coherent and efficient method for automated object detection and recognition in high-resolution remote sensing imagery, including the mix-grained data fusion, data augmentation and the model ensemble with class-specific NMS. It has the following advantages.

1. By adopting the mix-grained data fusion strategy to merge FAIR1M-2.0 [2] with the competition dataset, the champion’s solution increases the number of annotated data and is able to learn semantic information from more samples that is beneficial for this task, which helps enhance the generalization of the learned detection model.
2. The solution applies several effective data augmentation strategies to address the angle arbitrariness, long-tail distribution and color space inconsistency problems in the remote sensing detection task.
3. The solution proposes the class-specific NMS to exploit the merits of both single- and two-stage detectors for improving the detection capability of the ensemble. It makes the most of the detection results of the two models by applying different NMS strategies for different-sized categories.
4. The champion team achieves the top-1 score in both the preliminary and final rounds of the competition, demonstrating the robustness of the method. Meanwhile, the solution is concise and easy to reproduce.

### CONCLUSION AND FUTURE DIRECTION

The competition aims to stimulate the development of remote sensing detection and recognition algorithms based on deep learning technology. The participating teams have developed a number of novel and effective solutions, which are beneficial for this field. Despite the significant success of the competition, there are still some possible directions for future research.

1. For objects with different scales in remote sensing scenes, the champion’s solution employs the model ensemble strategy to enhance the scale robustness of the model, which also brings pressure on the speed of inference. The design of a more capable single detection model that is robust to the object size can be studied to further optimize the model efficiency.
2. In addition to CNN-based methods, transformer-based approaches, which have demonstrated outstanding performance in natural object detection tasks, might be explored for the remote sensing field.
3. Most of the existing models with superior performance are fully supervised. Considering the high cost of data annotation, methods based on few-shot learning can be developed in the future to get rid of the dependence on labeled data.
4. The resolution of the optical satellite images has reached 0.3 m. It is possible to identify the fine-grained categories of objects from the remote sensing scenes. Algorithms with fine-grained object recognition capability will be useful in some practical applications in the future.

## Supplementary Material

nwad122_Supplemental_File
