# Supplementary material for: Automated object recognition in high-resolution optical remote sensing imagery
Source: Natl Sci Rev. 2023 May 4;10(6):nwad122. doi: 10.1093/nsr/nwad122 (PMC10265956; doi:10.1093/nsr/nwad122)
Supplement: nwad122_Supplemental_File [file nwad122_supplemental_file.pdf]

PERSPECTIVE

National Science Review
XX: output, Year
doi: https://doi.org/10.1093/nsr/XXXX

PHYSICS

Special Topic: AI Algorithms and Cases : To Energize Digital Economy

Automated Object Recognition in High-resolution Optical Remote Sensing Imagery

Yazhou Yao[1,†], Tao Chen[1,†], Hanbo Bi[2,3,4], Xinhao Cai[1], Gensheng Pei[1], Guoye Yang[5], Zhiyuan Yan[2,3,4,*], Xian Sun[2,3,4,*], Xing Xu[6,8] and Hai Zhang[7,8,*]

[1]School of Computer Science and Engineering, Nanjing University of Science and Technology, Nanjing 210094, China; [2]Aerospace Information Research Institute, Chinese Academy of Sciences, Beijing 100190, China; [3]School of Electronic, Electrical and Communication Engineering, University of Chinese Academy of Sciences, Beijing 100049, China; [4]Key Laboratory of Network Information System Technology (NIST), Aerospace Information Research Institute, Chinese Academy of Sciences, Beijing 100190, China; [5]Department of Computer Science and Technology, Tsinghua University, Beijing 100084, China; [6]School of Computer Science and Engineering, University of Electronic Science and Technology of China, Chengdu 611731, China; [7]School of Mathematics, Northwest University, Xi'an 710127, China; [8]Pazhou Laboratory Huangpu, Guangzhou 510700, China

[*]**Corresponding authors.** Email: yanzy@aircas.ac.cn; sunxian@aircas.ac.cn; zhanghai@nwu.edu.cn.
[†]Equally contributed to this work..

Received: XX XX Year;
Revised: XX XX Year;
Accepted: XX XX Year

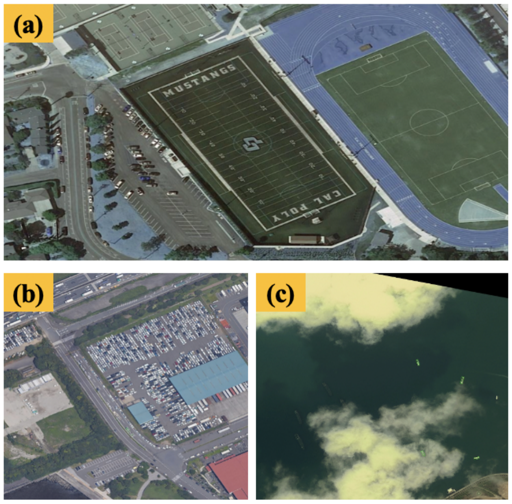

Figure S1. The challenges for automated object detection and recognition in remote sensing images. (a) Large variation in object sizes. (b) Dense distribution of small objects. (c) Low signal-to-noise ratio.

Table S1. Comparison of mAP of the first 8 teams in the competition

Team Rank	preliminary	final
1	**81.16**	**74.16**
2	81.11	73.94
3	79.07	72.9
4	78.65	72.75
5	78.09	71.39
6	78.62	71.33
7	78.92	71.03
8	78.38	70.26

Table S2. Element-wise component analysis

Data Aug	Data Fusion	CS NMS	mAP
			75.63
✓			77.52
✓	✓		80.15
✓	✓	✓	**81.16**

Table S3. Comparison of model size and inference speed on a remote sensing image with 1024 × 1024 pixels

Model	Size	Time
Single-stage detector	56M	0.17s
Two-stage detector	105M	0.35s
Ensemble	161M	0.52s

© The Author(s) Year. Published by Oxford University Press on behalf of China Science Publishing & Media Ltd. This is an Open Access article distributed under the terms of the Creative Commons Attribution License (http://creativecommons.org/licenses/by/4.0/), which permits unrestricted reuse, distribution, and reproduction in any medium, provided the original work is properly cited.

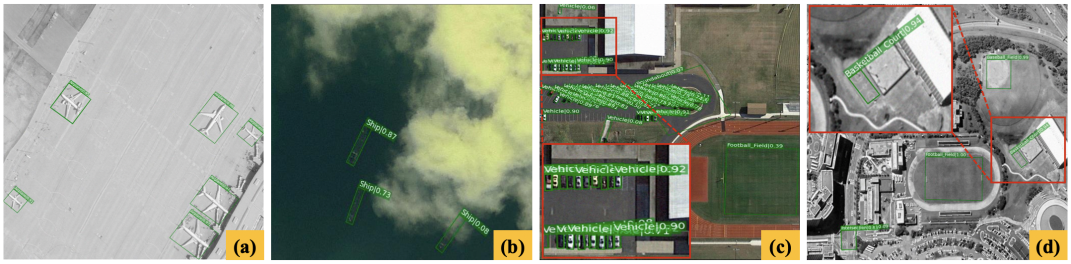

Figure S2. Visualization of the detection results from the champion's solutions. (a) Airplane detection results with different rotations. (b) Ship detection results under the cover of clouds. (c) Small vehicle detection results with dense distribution. (d) Detection results with multi-scale objects.
